# Prevalence of Dysexecutive Symptoms in High School Students during the COVID-19 Pandemic

**DOI:** 10.3390/ijerph192315641

**Published:** 2022-11-24

**Authors:** Guillermo Alonso Cervantes-Cardona, Adriana Nápoles-Echauri, Nicolas Alonso-Estrella, Francisco Javier Hernández-Mora, Enrique Cervantes-Pérez, Gabino Cervantes-Guevara, Benjamín García-Reyna, Francisco José Barbosa-Camacho, Noelia Esthela López-Bernal, Jonathan Matías Chejfec-Ciociano, Clotilde Fuentes-Orozco, Tania Abigail Cueto-Valadez, Andrea Estefanía Cueto-Valadez, Irma Valeria Brancaccio-Pérez, Mario Jesús Guzmán-Ruvalcaba, Jesús Oswaldo Vega-Gastelum, Alejandro González-Ojeda

**Affiliations:** 1 Departamento de Disciplinas Filosófico, Metodológicas e Instrumentales, Centro Universitario de Ciencias de la Salud, Universidad de Guadalajara, Guadalajara 44340, Jalisco, Mexico; 2 Escuela Vocacional, Sistema de Educación Media Superior Universidad de Guadalajara, Guadalajara 44430, Jalisco, Mexico; 3 Departamento de Clínicas de la Reproducción Humana, Crecimiento y Desarrollo Infantil, Centro Universitario de Ciencias de la Salud, Universidad de Guadalajara, Guadalajara 44348, Jalisco, Mexico; 4 Departamento de Medicina Interna Hospital Civil de Guadalajara Fray Antonio Alcalde, Centro Universitario de Ciencias de la Salud, Universidad de Guadalajara, Guadalajara 44280, Jalisco, Mexico; 5 Departamento de Bienestar y Desarrollo Sustentable, Centro Universitario del Norte, Universidad de Guadalajara, Colotlán 46200, Jalisco, Mexico; 6 Unidad Médica de Alta Especialidad Pediatría, Centro Médico Nacional de Occidente, Instituto Mexicano del Seguro Social, Guadalajara 44280, Jalisco, Mexico; 7 Unidad de Investigación Biomédica 02, Hospital de Especialidades del Centro Médico Nacional de Occidente, Instituto Mexicano del Seguro Social, Guadalajara 44349, Jalisco, Mexico

**Keywords:** cognitive development, neuropsychology, psychometrics, isolation, emotional response

## Abstract

This is an observational cross-sectional study designed to ascertain the prevalence and severity of dysexecutive symptoms in high school students during the COVID-19 pandemic. The validated Spanish version of the Dysexecutive Questionnaire (DEX) was used. A total of 2396 participants aged 14–22 years were included. Our sample yielded a mean DEX scale score of 28.14 ± 17.42. By the DEX classification, 889 (37.1%) students achieved optimal scores, 384 (16%) reported mild dysexecutive symptoms, 316 (13.2%) reported moderate dysexecutive symptoms, and 807 (33.7%) reported strong dysexecutive symptoms. We found a significant difference between those with and those without employed mothers, with the former scoring higher (*p* = 0.004), the same as those with both parents employed (*p* = 0.004). Adolescents face emotional susceptibility and changes in their family, social, and educational environment related to isolation, resulting in altered emotional responses and social interaction.

## 1. Introduction

Adolescence is a critical developmental stage marked by numerous physical, social, and cognitive changes. The considerable optimization of internal mental processes, such as the executive functions, is one of the most essential growth milestones of youth [1].

The executive function reflects the cognitive processes underlying goal-directed behaviors, emotional responses, and social interaction. It is essential for building resilience to cope with stressful life events. When these functions are altered, or their development is delayed, adolescents may experience problems in managing their emotions, as well as in developing cognitive or emotional abilities that allow for the planning and execution of functional behavior [2]. The executive function deficits manifest in the form of disorganized behavior in different domains of human functioning, including the disruption of self-awareness, interpersonal communication, professional activity, and everyday life [3]. A drug or alcohol addiction, cranioencephalic trauma, neurological infection or diseases, and emotional distress are all factors that may affect the executive function in healthy teens. This prefrontal dysfunction is not always evident in neuropsychological tests, but it has been observed in the management of daily occupations [1].

Several instruments are now available to assess neurocognitive impairment. The Dysexecutive Questionnaire (DEX) is an important instrument for the study of executive functions such as inhibition, intention, social regulation, and abstract problem-solving [4,5]. The DEX was designed to be a qualitative measure of symptoms in everyday life; nonetheless, its use in a nonclinical population may be a good measure of the frontal function, and its clinical utility has prompted numerous researchers to investigate its quantitative features. Chan et al. [4] proposed that the DEX may provide a screening of the executive functions of patients without reported frontal lobe damage, psychiatric comorbidities, or personality disorders.

During the COVID-19 pandemic, children and adolescents worldwide had a higher prevalence of depression and anxiety [6]. Furthermore, research has revealed that youths living in areas with poor outcomes experience more distress [7]. These variables have been linked to changes in the executive function [3,8].

It is also worth noting that frontal lobe damage has been described as a COVID-19 consequence [9]. Previous research has found that patients who have had COVID-19 and developed encephalopathy during the infection period may have altered cognitive functions [10]. It was also reported that patients who had symptoms including fatigue and neurological impairment had a decline in their executive functions [11,12]. Moreover, there may be a relationship between acute respiratory distress syndrome, which can alter the cognitive function of these patients, especially those who need mechanical ventilation, and the development of neurocognitive symptoms in the long term [13,14]. 

The purpose of this study was to determine the prevalence of dysexecutive symptoms in high school students during the COVID-19 pandemic. Research indicates that the mental health and neurological development of our children and adolescents can be significantly impacted by the direct influence of COVID-19 on behavior as a result of social isolation and its psychological and neurological ramifications during and after infection [15,16,17,18].

## 2. Materials and Methods

### 2.1. Objective

The aim of this study was to identify the prevalence and severity of dysexecutive symptoms in high school students during the COVID-19 pandemic.

### 2.2. Design

This is an observational cross-sectional study in which an anonymous online survey was conducted between September and October 2021. The validated Spanish language version of the DEX inventory [19] was used. We asked the participants about their gender and age, whether they or a family member had been infected with COVID-19, whether any of the infected family members had died, and whether one or both of their parents were employed.

### 2.3. Sample

This study was conducted between September and October 2021 in a public high school in Guadalajara, Jalisco, Mexico. The sample size was calculated following a study by Pedrero-Pérez that reported a mean DEX score of a nonclinical sample of 37.52 ± 7.78, while the clinical sample presented a mean DEX score of 50.69 ± 14.39. A minimum sample size of 84 individuals was calculated using an alpha error of 0.05 and a beta error of 0.10. This study included 2396 students aged from 14 to 22 years with an average age of 17 years. The students were asked to participate. After their consent was obtained, the survey was administered, and the responses were anonymous.

### 2.4. Instruments

The validated Spanish version of DEX was used for this study [20]. It consists of 20 items that measure a range of dysexecutive symptoms with five-point Likert response scales ranging from 0 (never) to 4 (very often), with higher scores indicating a greater severity of executive functioning problems. The DEX can be used to assess four factors of dysexecutive syndrome: inhibition (items 2, 5, 12, 13, 14, and 15), intention, (items 17, 18, and 19), social regulation (items 4, 7, 8, 11, 16, and 20), and abstract problem-solving (items 1, 3, 6, and 9). The scale showed a great internal reliability in the four factors: inhibition (α = 0.798), intention (α = 0.793), social regulation (α = 0.813), and abstract problem-solving (α = 0.805).

The cut-off scores were classified as follows [19]:

- <20: optimal performance.
- 20–27: mild symptoms.
- 28–35: moderate symptoms.
- ≥36: strong symptoms.

The recruitment team went to the high school’s classrooms and invited the students to answer the survey, outlining the study’s purpose and what their involvement would entail. Students were given a link to the online survey once they had consented to it. A time limit of 15 min per classroom was set, and instructions on how to complete the survey were provided. The information was gathered in a database after the participants were acknowledged.

### 2.5. Data Analysis

The statistical analysis was conducted using IBM SPSS Statistics Version 25. The variables were evaluated using central tendencies, such as the mean, standard deviation, and frequencies. The study’s outcome variables were the DEX scale mean scores and the subscales mean scores (inhibition, intention, social regulation, and abstract problem-solving). The inferential analysis of the categorical variables (gender [female/male], age groups [14–16/17–22 years], the COVID-19 infection status [yes/no], if any family members has been infected [yes/no], if any of the infected family members had died from COVID-19 [yes/no], and whether one or both parents were employed were the study’s dependent variables [yes/no]) was performed using the chi-squared test. For the quantitative variables (DEX scale and subscale scores), Student’s t-test or Mann–Whitney U tests were performed depending on the data distribution. All the variables were assessed with Levene’s Test for Equality of Variances, finding that most variables had a parametric distribution. When a non-parametric distribution was found, we performed the Mann–Whitney U test. A probability value of <0.05 was considered to be statistically significant.

## 3. Results

A total of 2396 participants were included in this study. Their demographic data are presented in Table 1. We divided the participants into two age groups: 14–16 years (*n* = 1227, 51.2%) and 17–22 years (*n* = 1169, 48.8%). Our sample yielded a mean DEX scale score of 28.14 ± 17.42. The mean factor scores were 9.87 ± 6.28 for inhibition, 5.10 ± 3.32 for intention, 8.65 ± 5.63 for social regulation, and 4.52 ± 3.88 for abstract problem-solving. By the DEX classification, 889 (37.1%) students obtained optimal scores, 384 (16%) reported mild dysexecutive symptoms, 316 (13.2%) reported moderate dysexecutive symptoms, and 807 (33.7%) reported strong dysexecutive symptoms.

When we compared the DEX scale mean scores by gender, female participants had a higher mean score than male participants. This was also true when we compared the inhibition, intention, social regulation, and abstract problem-solving scores. When comparing age groups, we found that those aged below 18 years scored lower than older people across all factors, except for abstract problem-solving. However, none of the factors showed a statistical significance. Similarly, the students in semesters 1–3 had higher scores than those in semesters 4–6. This difference was statistically significant in all factors.

Compared with uninfected peers, students who were infected with COVID-19 had significantly higher scores on all factors. A Mann–Whitney U test was conducted to determine whether there was a difference in the DEX scale scores: the results indicated significant differences between the groups in the DEX mean scores (U = 165093.50, *p* = 0.007), inhibition scores (U = 664155, *p* = 0.011), and abstract problem-solving scores (U = 166623, *p* = 0.011). In addition, students with family members infected with COVID-19 scored significantly higher on all the four factors than those without. We found statistically significant differences across all the variables, except the social regulation (DEX mean score [t = 2.89, *p* = 0.004], inhibition [U = 664155, *p* = 0.006], intention [t = 2.83, *p* = 0.005], and abstract problem-solving [t = 2.64, *p* = 0.008]. However, there was no statistically significant difference between students with family members who had survived COVID-19 and those who had lost family members to the disease.

We found that students with working mothers scored significantly higher on inhibition, intention, social regulation, and abstract problem-solving than those whose mothers were not employed. This difference was statistically significant. Comparing the scores of students with and without working fathers revealed no statistically significant differences in any of the variables. The mean DEX scores and all the factor scores are presented in Table 2. 

The DEX performance scores showed similar proportions across all the groups. Most participants fell between the extremes of optimal performance and strong symptoms. We found a significant difference only between those with employed mothers and those without, with higher scores among those with employed mothers (*p* = 0.004), similar to students with both parents employed (*p* = 0.004). The overall distribution is shown in Table 3.

## 4. Discussion

Although the DEX was developed with frontal-lobe dysfunction in mind, it can be utilized for a variety of patients who have had a brain injury, schizophrenia, a drug addiction, or a condition affecting the prefrontal cortex in general [5].

Owing to the process of maturation and self-knowledge by which people discover and experience new situations, the years preceding puberty can be difficult [21]. Although it has been discovered that the likelihood of developing dysfunctional symptoms increases dramatically as the brain ages [22], the process of integrating adaptive and cognitive tools is critical to social functional behavior during adolescence [23]. This could explain why the younger students had higher mean scores than the older students, as they are still developing functional executive processes.

Inconsistent results on the DEX scores in nonclinical samples have been documented in the scientific literature. Our sample scored higher than a clinical and non-clinical sample collected prior to the COVID-19 pandemic [3,24]. However, the scores were much lower than those reported by Pedrero et al. when comparing to another population [20]. The overall DEX scale and subscale scores of the students affected by COVID-19 were considerably higher than those of healthy students. This could be explained by the findings that COVID-19 patients exhibit psychological symptoms such as anxiety, depression, and stress, which all contribute to executive dysfunction [18,25,26]. COVID-19 has shown a direct association with the central nervous system symptoms. Studies and case reports emerging from China, France, and various other parts of the world have revealed a spectrum of neurological symptoms ranging from simple headaches to more serious signs, such as impairments in cognition, memory, or information processing [13,27,28].

The pathophysiology of COVID-19 can be explained in terms of an invasion by SARS-CoV-2 into cells in the host body, resulting in an inflammatory response and symptoms. The blood–brain barrier is compromised by the systemic inflammation associated with a SARS-CoV-2 infection, which severely disrupts brain homeostasis and causes neuronal cell death [29]. Subsequently, an infection of the brain stem may affect the chemosensory neural cells associated with a respiratory and cardiovascular regulation, as well as neurons of the respiratory center. The combination of hypoxia with existent neuro-inflammation causes damage to the hippocampal and cortical areas, resulting in the neuropsychiatric effects of the virus [9,30].

Our comparisons show that female students reported significantly higher scores on all four factors than males. This is interesting, as in other studies, male participants received higher DEX scores; however, the differences between males and females were not significant on any subscale, or in the overall DEX score [19,20].

Interestingly, there was no effect on the executive functions of adolescents when their fathers were employed, but there was a significant effect on the development of dysexecutive symptoms when their mothers or both parents were employed. Similar to the findings reported by Bacikova-Sleskova, where adolescents reported negative perceptions of their unemployed fathers, there was no negative perception of their unemployed mothers [31]. According to research, maternal affection can reduce the incidence of anxious and depressive symptoms in the younger population [32,33]. This is intriguing because, depending on the family’s customs or economic situation, the economic strain can determine the division of the employment role between parents. Mexican mothers traditionally stay at home, and fathers support the family economically, which may explain why the advent of working mothers may cause teens to perceive a change in their family dynamics. Some studies have examined the extent to which there were protective benefits from incorporating mindfulness training into a mentoring program to buffer adolescents against negative pandemic health effects [27]. Similarly, a good night’s sleep has been linked to a reduction in executive dysfunction in adolescents [34]. It is recommended that adolescents sleep between eight and nine hours per night to maintain a healthy behavioral and cognitive development [35].

It is crucial to emphasize certain limitations of this study. First, the DEX is mainly used for clinical patients with an anatomical brain alteration or disease for which the presenting symptoms are pathological. In addition, the version of the inventory used was the Spanish language version, however, this is not a Mexico-specific version. Second, students from only one school responded, so we cannot infer that students from other schools would receive the same scores, as each school differs in context, costs, and region. Third, the school represents a low economic class, so we cannot predict that other students of the same age from another socioeconomic background would think or feel the same way. We noted in this study that age did not show a statistically significant *p*-value and that certain ages in life could favor neurological symptoms or a prefrontal cortex degeneration owing to physiological changes.

## 5. Conclusions

The COVID-19 pandemic has led to social isolation. Adolescents in particular face emotional susceptibility, as well as changes in their family, social, and educational environments related to isolation, resulting in altered emotional responses and social interaction. The analysis results identified that the DEX is a valuable instrument for assessing adolescent dysexecutive functions, as it considers both clinical patients—that is, those with a diagnosis of brain damage—and nonclinical patients without brain damage. The present study provides evidence of the impaired executive functioning associated with the COVID-19 pandemic.

## Figures and Tables

**Table 1:** Participants’ demographic characteristics.

Demographic Characteristics	Sample
Age (mean, SD)	16.53 ± 1.06
Gender (*n*, %)	
Female	1598 (66.7%)
Male	798 (33.3%)
Age groups (*n*, %)	
14–16	1227 (51.2%)
17–22	1169 (48.8%)
COVID-19 infection (*n*, %)	
Yes	166 (6.9%)
No	2230 (93.1%)
Family member infected (*n*, %)	
Yes	1344 (56.1%)
No	1052 (43.9%)
Family member deceased (*n*, %)	
Yes	185 (13.7%)
No	1159 (86.2%)
Employed mother (*n*, %)	
Yes	1525 (63.6%)
No	871 (36.4%)
Employed father (*n*, %)	
Yes	2144 (89.5%)
No	252 (10.5%)
Both parents employed (*n*, %)	
Yes	1341 (56%)
No	1055 (44%)

**Table 2:** DEX-Sp scale and subscales scores distribution between groups.

Gender	DEX-Sp Mean Score	t Score	*p*-Value	Inhibition	t Score	*p*-Value	Intention	t Score	*p*-Value	Social Regulation	t Score	*p*-Value	Abstract Problem-Solving	t Score	*p*-Value
Female	29.09 ± 17.50	−3.79	0.001	10.12 ± 6.29	−2.74	0.006	5.42 ± 3.37	−6.55	0.001	8.90 ± 5.60	−3.12	0.002	4.64 ± 3.95	−2.05	0.040
Male	26.23 ± 17.11			9.37 ± 6.25			4.48 ± 3.12			8.14 ± 5.65			4.29 ± 3.72		
Age groups															
14–16	28.75 ± 17.18	1.76	0.078	9.95 ± 6.27	0.61	0.541	5.23 ± 3.29	1.88	0.059	8.87 ± 5.52	1.92	0.054	4.66 ± 3.88	1.73	0.082
17–22	27.50 ± 17.65			9.79 ± 6.30			4.97 ± 3.34			8.42 ± 5.73			4.38 ± 3.88		
COVID-19 infection															
Yes	31.65 ± 19.02	--	0.007 *^‡^*	11.07 ± 7.10	--	0.011 *^‡^*	5.68 ± 3.44	2.32	0.020	9.54 ± 6.16	2.10	0.035	5.27 ± 4.29	--	0.011 *^‡^*
No	27.88 ± 17.27			9.78 ± 6.21			5.06 ± 3.31			8.58 ± 5.58			4.47 ± 3.84		
Family member infected															
Yes	29.05 ± 17.43	2.89	0.004	10.18 ± 6.37	--	0.006 *^‡^*	5.27 ± 3.30	2.83	0.005	8.85 ± 5.64	1.92	0.054	4.71 ± 3.92	2.64	0.008
No	26.98 ± 17.33			9.47 ± 6.16			4.89 ± 3.33			8.40 ± 5.59			4.29 ± 3.81		
Family member deceased															
Yes	28.78 ± 17.61	−1.24	0.212	10.12 ± 6.48	--	0.339	5.20 ± 3.32	−1.73	0.083	8.78 ± 5.69	0.19	0.363	4.67 ± 3.39	−0.87	0.384
No	30.50 ± 16.22			10.60 ± 5.65			5.66 ± 3.19			9.18 ± 5.35			4.94 ± 3.86		
Employed Mother															
Yes	28.93 ± 17.40	2.96	0.003	10.13 ± 6.31	2.66	0.008	5.29 ± 3.34	3.55	0.001	8.89 ± 5.61	2.75	0.006	4.66 ± 3.87	2.18	0.029
No	26.74 ± 17.37			9.42 ± 6.22			4.79 ± 3.26			8.23 ± 5.64			4.30 ± 3.88		
Employed Father															
Yes	28.10 ± 17.39	−0.27	0.781	9.86 ± 6.29	−0.15	0.875	5.11 ± 3.32	0.15	0.878	8.63 ± 5.63	−0.46	0.64	4.50 ± 3.87	−0.74	0.455
No	28.43 ± 17.66			9.93 ± 6.29			5.07 ± 3.34			8.80 ± 5.59			4.70 ± 3.95		
Both parents employed															
Yes	28.99 ± 17.31	2.68	0.007	9.91 ± 6.20	2.46	0.014	5.30 ± 3.33	3.18	0.001	8.89 ± 5.59	2.37	0.018	4.65 ± 3.86	1.84	0.066
No	27.06 ± 17.50			9.24 ± 6.12			4.48 ± 3.29			8.34 ± 5.65			4.36 ± 3.89		

Notes: *‡*: *p* value was obtained using the Mann–Whitney U test.

**Table 3:** DEX-Sp Classification scores distribution between groups.

Gender	Optimal Performance	Mild Symptoms	Moderate Symptoms	Strong Symptoms	X^2^	*p*-Value
Female	563 (35.2%)	257 (16.1%)	212 (13.3%)	566 (35.4%)	8.867	
Male	326 (40.9%)	127 (15.9%)	104 (13%)	241 (30.2%)		0.031
Age groups						
14–16	433 (35.3%)	194 (15.8%)	179 (14.6%)	421 (34.3%)	6.337	
17–22	456 (39%)	190 (16.3%)	137 (11.7%)	389 (33%)		0.096
COVID-19 Infection						
Yes	53 (31.9%)	27 (16.3%)	21 (39.2%)	65 (39.2%)	2.921	
No	836 (37.5%)	357 (16%)	295 (13.2%)	742 (33.3%)		0.404
Family member infected						
Yes	476 (35.4%)	218 (16.2%)	170 (12.6%)	480 (35.7%)	6.852	
No	413 (39.3%)	166 (15.8%)	146 (13.9%)	327 (31.1%)		0.077
Family member deceased						
Yes	423 (36.2%)	195 (16.7%)	137 (11.7%)	412 (35.2%)	14.053	
No	56 (30.3%)	26 (14.1%)	33 (17.8%)	70 (37.8%)		0.063
Employed mother						
Yes	528 (34.6%)	249 (16.3%)	200 (13.1%)	548 (35.9%)	13.536	
No	3651 (41.4%)	135 (15.5%)	116 (36.7%)	259 (29.7%)		0.004
Employed father						
Yes	794 (37%)	350 (16.3%)	281 (13.1%)	719 (33.5%)	1.389	
No	95 (37.7%)	34 (13.5%)	35 (13.9%)	88 (34.9%)		0.708
Both parents employed						
Yes	460 (34.3%)	223 (16.6%)	172 (12.8%)	486 (36.2%)	13.360	
No	429 (40.7%)	161 (15.3%)	144 (13.6%)	321 (30.4%)		0.004

## Data Availability

The datasets used and/or analyzed during the current study are available from the corresponding author upon reasonable request.
